# Application of convex hull analysis for the evaluation of data heterogeneity between patient populations of different origin and implications of hospital bias in downstream machine-learning-based data processing: A comparison of 4 critical-care patient datasets

**DOI:** 10.3389/fdata.2022.603429

**Published:** 2022-10-31

**Authors:** Konstantin Sharafutdinov, Jayesh S. Bhat, Sebastian Johannes Fritsch, Kateryna Nikulina, Moein E. Samadi, Richard Polzin, Hannah Mayer, Gernot Marx, Johannes Bickenbach, Andreas Schuppert

**Affiliations:** ^1^Institute for Computational Biomedicine, RWTH Aachen University, Aachen, Germany; ^2^Joint Research Center for Computational Biomedicine, RWTH Aachen University, Aachen, Germany; ^3^SMITH Consortium of the German Medical Informatics Initiative, Leipzig, Germany; ^4^Department of Intensive Care Medicine, University Hospital RWTH Aachen, Aachen, Germany; ^5^Juelich Supercomputing Centre, Forschungszentrum Juelich, Juelich, Germany; ^6^Systems Pharmacology and Medicine, Bayer AG, Leverkusen, Germany

**Keywords:** dataset-bias, data pooling, ARDS, convex hull (CH), generalization error

## Abstract

Machine learning (ML) models are developed on a learning dataset covering only a small part of the data of interest. If model predictions are accurate for the learning dataset but fail for unseen data then generalization error is considered high. This problem manifests itself within all major sub-fields of ML but is especially relevant in medical applications. Clinical data structures, patient cohorts, and clinical protocols may be highly biased among hospitals such that sampling of representative learning datasets to learn ML models remains a challenge. As ML models exhibit poor predictive performance over data ranges sparsely or not covered by the learning dataset, in this study, we propose a novel method to assess their generalization capability among different hospitals based on the convex hull (CH) overlap between multivariate datasets. To reduce dimensionality effects, we used a two-step approach. First, CH analysis was applied to find mean CH coverage between each of the two datasets, resulting in an upper bound of the prediction range. Second, 4 types of ML models were trained to classify the origin of a dataset (i.e., from which hospital) and to estimate differences in datasets with respect to underlying distributions. To demonstrate the applicability of our method, we used 4 critical-care patient datasets from different hospitals in Germany and USA. We estimated the similarity of these populations and investigated whether ML models developed on one dataset can be reliably applied to another one. We show that the strongest drop in performance was associated with the poor intersection of convex hulls in the corresponding hospitals' datasets and with a high performance of ML methods for dataset discrimination. Hence, we suggest the application of our pipeline as a first tool to assess the transferability of trained models. We emphasize that datasets from different hospitals represent heterogeneous data sources, and the transfer from one database to another should be performed with utmost care to avoid implications during real-world applications of the developed models. Further research is needed to develop methods for the adaptation of ML models to new hospitals. In addition, more work should be aimed at the creation of gold-standard datasets that are large and diverse with data from varied application sites.

## Introduction

Driven by giant leaps in compute performance, the availability of huge datasets, and new algorithms for the training of deep neural networks (DNN), Machine Learning (ML) has seen a renaissance during the last 10 years. Today, ML approaches help us discover patterns in large swaths of data, predominantly on an automated or semi-automated basis. They have revolutionized how we process images, video, and text. The primary advantage of ML when compared to traditional modeling approaches for the input-output behavior of complex systems is the unbiased learning from data without a priori knowledge about the system to be learned (black-box modeling approach). Mathematically, ML algorithms are designed as universal machines mapping a high dimensional input space onto a low dimensional output space up to an order of error without restrictions. The algorithms enable unrestricted learning by a modeling strategy with a priori unrestricted complexity of the model, e.g., expressed by the unrestricted number of parameters to be adapted to the data. For large classes of functions, ML algorithms, e.g., neural networks, provide superior approximation performance compared to all linear series expansions (Barron and Klusowski, 2018). Recently, the equivalence of DNN learning with wavelet-based approximations indicated the superior performance of DNN for applications with close association with image recognition and time-series analysis (Mallat, 2016).

Data-driven models, such as ML methods, aim to represent systems solely from available measurement data. Hence, a critical conceptual issue of such models is their limited performance in the case of extrapolation into data regions sparsely covered by the data samples used for learning the model. These models handle test data better if they come from the same dataset used for training and generalize worse on the data obtained from other sources (Torralba and Efros, 2011; AlBadawy et al., 2018; Pooch et al., 2019). Model performance drops if data used to train and test a model come from different distributions. This difference is referred to as a domain shift (Pooch et al., 2019). Unless strong assumptions are posed on the learned function, data-driven models, not depending on the output to be predicted, can only be valid in regions where they have sufficiently dense coverage of training data points, which is referred to as the validity domain (Courrieu, 1994). This can be approximated by the convex hull spanned by the data, which represents an upper bound of the validity domain for any ML application. The convex hull (CH) of a set of data points is defined as the smallest polytope with dimensionality equal to the number of attributes containing the points in such a way that every straight line connecting a pair of points lies inside the polytope (Graham, 1972; Shesu et al., 2021). One approach to estimate the ability of a model to generalize is to consider the CH of the points used in a training set. Generalization tends to fail with the increase in the distance of a new point to the CH of the training set (Zhou and Shi, 2009). Therefore, the coverage of the CH of a test set by the CH of a training set represents an upper bound for the generalization ability of any ML-based model. In the case of learning from different populations, the mutual coverage of the convex hulls can serve as a measure for the sufficient similarity of heterogeneous populations enabling the first estimate for the reliability of the generalization of ML models. Hence, one possible approach to examine different populations for homogeneity concerning the predictive performance of ML models is to perform a convex hull analysis of the available data to be used for training and prediction, respectively (Ostrouchov and Samatova, 2005; Zhou and Shi, 2009).

However, even if the convex hulls of training and test sets intersect to a large extent, there might be differences in the underlying distributions of some parameters. For instance, when data of one dataset lay in a region which shows a low density of samples in the other dataset. An extreme example is a dataset consisting of two clusters of data apart from each other; the convex hull envelopes all dataset values, including the space between them. If the majority of samples of the second dataset fall inside the gap area between the two clusters, the generalization capacity of a model will be impaired, as there is not enough training data in that region. Although the intersection values are high, in this case, it does not allow us to judge the generalization ability of the trained model. Therefore, the CH analysis provides necessary, but not sufficient conditions for a proper generalization of ML models.

Consequently, a second step in the analysis is needed to investigate datasets for diverging underlying distributions. If there are no such differences, two datasets form a homogeneous population and are indistinguishable, otherwise, it would be possible to differentiate the datasets. Therefore, if ML classifiers can identify the origin of a drawn sample with high accuracy, we postulate that there are diverging underlying distributions of parameters forming different areas with a high density of samples in two datasets. Thus, training an ML model in one dataset and applying it in the other one would mean interpolation into areas sparsely covered by training data and could impair the generalization of respective models. However, ML methods do not provide the direction of impaired generalization (i.e., model trained on one dataset and applied in the other one and vice versa).

In contrast, CH analysis provides a model-agnostic a priori data assessment and more importantly direction of impaired generalization. The CH of one dataset may completely cover the CH of the other dataset, meaning no restrictions for generalization from the CH perspective. However, in the opposite case (the second dataset covering the first one) the CH coverage may be modest suggesting generalization issues once models developed in the second dataset will be applied to the first dataset. Furthermore, the CH analysis proposed in this paper is computationally inexpensive and is an order of magnitude faster than ML methods. Therefore, we suggest an application of the CH method for universal generalization assessment supported by the application of ML methods to reveal the scope of differences in underlying distributions. Combining the results of these 2 methods, one receives a complete vision of potential generalization issues.

In medicine, the application of ML promises to provide solutions for unmet needs in clinical practice which have partly been hampered by a missing mechanistic understanding of the underlying processes. Medical applications, like an early diagnosis of rare or complex diseases, optimization of therapeutic strategies or the surveillance of patients, and resource planning are expected to benefit from the advantages of ML significantly (Komorowski et al., 2018; Miotto et al., 2018; Shillan et al., 2019; Ghassemi et al., 2020). However, despite promising results for image-analysis-based medical applications or time-series monitoring (Arcadu et al., 2019; Tomasev et al., 2019), the superiority of DNN when compared to traditional approaches has not been proven yet (Chen et al., 2019). Moreover, it has been demonstrated that the design and integration of complex data analytics workflows play a key role in the performance of ML algorithms in biomedical applications (Schatzle et al., 2020). The realization of the promises of ML in medicine requires further innovations in a huge variety of challenges, ranging from data availability and learning strategies up to the integration of a priori knowledge into ML setup (Frohlich et al., 2018).

A highly crucial issue of ML application in medicine arises, when a model developed and trained on high-quality data of one hospital and showing good predictive performance, does not deliver adequate performance when applied to data of other hospitals. Hidden biases between hospitals could be caused by different admission strategies, guidelines for treatment, patients' baseline values, protocols on settings of medical support devices, or definitions of cut-off values (Kelliny et al., 2008). As an example, in 2019, Yan et al. built a simple data-driven model from electronic health records of 485 patients infected with SARS-CoV2 in the region of Wuhan, China (Yan et al., 2020). The authors claimed that their model could predict the outcome for patients with >90% accuracy using the values of three laboratory parameters only. However, the model failed to deliver the same high accuracy on patient datasets from hospitals in France, the USA, and the Netherlands (Barish et al., 2021; Dupuis et al., 2021; Quanjel et al., 2021).

In this work, we developed a pipeline for the comparison of populations and assessment of an ML model's generalization ability. First, we applied our CH analysis to find CH coverage values between datasets. Second, 4 types of ML models were trained to classify from which hospital a patient's sample originated. The performance of these models was assessed to judge, which datasets differ the most in terms of underlying data distributions. We applied our pipeline to 4 critical-care patient datasets of different origins: three datasets from German hospitals generated within the SMITH project (Marx et al., 2021) and the American “Medical Information Mart for Intensive Care” III (later referred to as MIMIC) dataset (Johnson et al., 2016). First, the pipeline was applied to every pair of hospitals to find mean CH coverages and performances of ML models for classification for a data source. Second, we investigated the applicability of the developed pipeline using the example of acute respiratory distress syndrome (ARDS)—a potentially life-threatening condition leading to respiratory insufficiency with possible multi-organ failure and fatal outcomes (Cochi et al., 2016; Raymondos et al., 2017). We showed that drops in the performance of models developed for the classification of ARDS on the first day in the Intensive Care Unit (ICU) were attributed to the poor intersection of convex hulls and to the large differences in underlying data distributions of corresponding hospitals.

## Methods

### Data

Three German hospitals (later referred to as Hosp A, Hosp B, and Hosp C) provided retrospective, fully anonymized data of ICU patients within the context of the use case “Algorithmic surveillance of ICU patients with acute respiratory distress syndrome” (ASIC) (Marx et al., 2021) of the SMITH consortium which is part of the German Medical Informatics Initiative. The ASIC project was approved by the independent Ethics Committee (EC) at the RWTH Aachen Faculty of Medicine (local EC reference number: EK 102/19). Patient inclusion criteria were age above 18 years and a cumulative duration of mechanical ventilation for at least 24 h. In addition, MIMIC was used as an independent dataset with different geographical origins. To identify the duration of invasive mechanical ventilation (MV) of patients from this dataset, a special MIMIC view was used^1^ Each patient's data included routinely charted ICU parameters collected over the whole ICU stay. The full list of parameters is given in Supplementary List S1. Data from all 4 sites were brought to the same units of measurement and were checked for consistency. The final number of patients in corresponding hospitals is given in Table 1.

**Table 1:** Clinical characteristics of the analyzed patient cohorts in four hospitals under consideration.

	**Hosp A**	**Hosp B**	**Hosp C**	**MIMIC**
Total number of patients, n (%)	13,067 (100)	2,976 (100)	1,368 (100)	7,683 (100)
Age, years (mean ± SD)	67.3 ± 14.5	67.3 ± 13.8	68.7 ± 13.0	64.1 ± 15.5
Male gender, n (%)	8,529 (65.3)	1,957 (65.8)	961 (70.2)	4,416 (57.5)
Length of stay ICU, days (mean ± SD)	17.3 ± 19.4	21.2 ± 20.1	18.7 ± 18.1	13.5 ± 12.4
Mortality, n (%)	3,742 (28.6)	828 (27.8)	608 (44.4)	1,277 (16.6)

Data for further analysis were prepared in the following way: first, the median values of routinely charted ICU parameters collected over the first day of ICU stay were extracted as features for the analysis. Features with values missing in more than 30% of patients were omitted. We considered features, that were present in all 4 hospitals after the data feature omission step. The final list of features (21 features overall) used in the analysis can be found in Supplementary List S2. Missing values of features were filled with the hospital-wide median value for that feature.

### Use case example: Classification for ARDS on the first day of treatment in ICU

To demonstrate the applicability of the developed pipeline, we considered the following typical use case of the application of ML models in healthcare: classification for a critical condition based on the first-day data. We used the presence of ARDS on the first day in the ICU as an endpoint for classification. The criteria for the diagnosis of an ARDS episode are defined in the Berlin criteria (ARDS Definition Task Force et al., 2012). However, in our use case scenario, only the criteria for oxygenation were taken into account. To be able to assess these criteria, only patients having parameters of MV [positive end-expiratory pressure (PEEP), a fraction of inspiratory oxygen (FiO_2_)] and blood gas analysis measurements [partial pressure of oxygen (PaO_2_)] during the first 24 h were selected.

ARDS patients were chosen based on ICD-10 codes (J80), where available. In the MIMIC database, ICD-9 coding system was used, which does not contain a specific code for ARDS. Therefore, the ARDS label was assigned to patients having ICD-9 codes for pulmonary insufficiency or respiratory failure (Reynolds et al., 1998): 5,185, 51,851, 51,852, 51,853, and 51,882. ARDS onset time was defined as a time point when the Horowitz index drops below 300 for the first time and stays below this threshold for at least 24 h. To ensure that information on the ARDS/non-ARDS status of patients is present in the data, only first-day ARDS patients were chosen as a case group. The Control group comprised all non-ARDS patients and patients with ARDS onset later than on the first day. A total number of day1-ARDS/non-ARDS patients in corresponding hospitals is given in Table 2.

**Table 2:** Number of day 1 non-ARDS/ARDS patients in hospitals.

**Hospital**	**Non-ARDS**	**ARDS (%)**
Hosp A	9,471	639 (6.3)
Hosp B	1,123	86 (7.1)
Hosp C	924	88 (8.7)
MIMIC	4,555	237 (4.9)

In this use case, we evaluated how a ML model trained in one hospital behaves in terms of performance if it is applied in another hospital. A Random Forest Classifier was trained in each of the four hospitals separately to classify ARDS and non-ARDS patients and tested in the other unseen hospitals. Performance in all datasets was assessed with ROC AUC.

### Convex hull analysis

**Figure 1:**
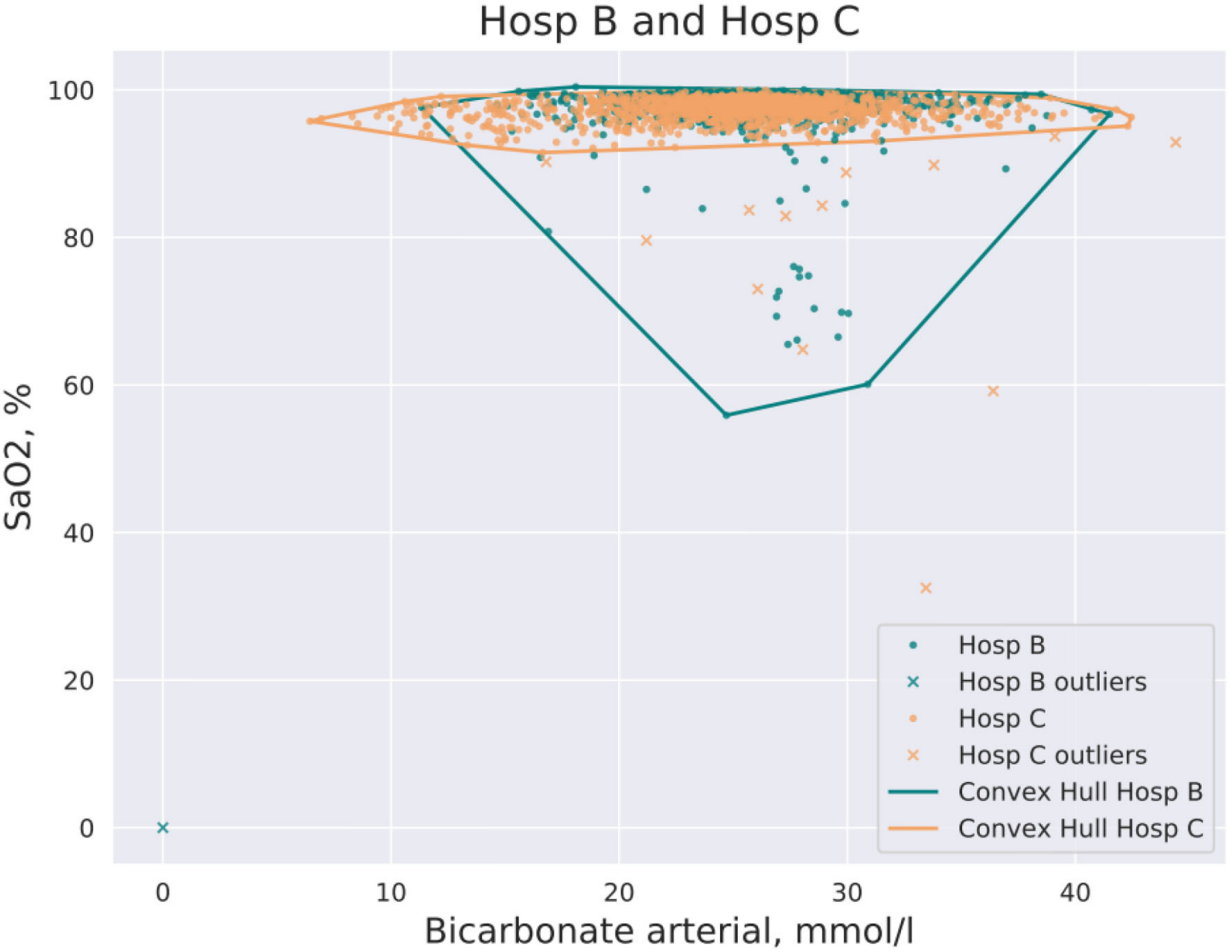
Example of CH intersection for the pair of hospitals (Hosp A, Hosp B) and the pair of features: SaO_2_ and bicarbonate. Some data points are filtered out by the DBSCAN method prior to the construction of the CH.

CH coverage for a new dataset was defined as the ratio of data points of a new dataset that lay inside of the CH of the initial dataset in the pair. An example of CH intersections for hospitals (Hosp B, Hosp C) and for the pair of features, arterial oxygen saturation (SaO_2_) and arterial bicarbonate, is shown in Figure 1. It should be noted that CH coverage is not a symmetric measure, i.e., CH coverage of Hosp A by Hosp B can differ from CH coverage of Hosp B by Hosp A. CH coverage for each feature combination was assessed in 2 dimensions, i.e., for each combination of pair of features the coverage of CH of one hospital was calculated for all other hospitals. For instance, if hospitals Hosp A and Hosp B were considered, for each pair of features, CH coverage of Hosp A by Hosp B and CH coverage of Hosp B by Hosp A were calculated. CH coverages were assessed using bootstrapping of underlying data (100 times). The equation for a CH coverage for a Hosp A by Hosp for a pair of features *(feature*^*i*^ *, feature*^*j*^*)*, where *i* and *j* denote feature indeces, is given by:


(1) $$\begin{array}{l} CH_{cov}\left(feature^{i},feature^{j}\right)\\ =\frac{\sum_{k\in HospA1}\left[\left(feature_{k}^{i},feature_{k}^{j}\right)\;\in \;CH_{ij}\left(HospB\right)\right]}{\sum_{k\in HospA1}} \end{array}$$

where *CH*_*ij*_*(Hosp B)* corresponds to the CH of the dataset of Hosp B in 2 dimensions *(feature*^*i*^*, feature*^*j*^*)*.

In higher dimensions intersections of CHs identified from datasets of sizes, which are usually available in single hospitals, tend to shrink even for datasets drawn from the same distribution due to the curse of dimensionality. Hence, we tested overlapping data by means of the overlaps of projections onto subspaces spanned by all combinations of 2 features. In case of overlapping CHs, the CHs of all projections will overlap as well. The opposite holds only in the case of homogeneous data distributions within the box in full data space spanned by the intersection of all projections. We assume that this is the case for real-world data available in healthcare and our approach delivers an acceptable approximation for the estimation of translational predictivity for practical use.

CH coverage for a feature was calculated as the median CH coverage value of all feature pairs that contain this feature:


(2) $$\begin{array}{l} CH_{cov}(feature^{j})=med(CH_{cov}(feature^{i},feature^{j}),\ldots ,\\ CH_{cov}(feature^{n},feature^{j})). \end{array}$$

Next, the distribution of CH coverages for all features was computed. Finally, mean CH coverages for each pair of hospitals were calculated as the mean CH coverage among all features:


(3) $$\begin{array}{l} CH_{cov}(Hosp\;A\;by\;Hosp\;B)=\frac{\sum_{i\in n}CH_{cov}(feature^{i})}{n} \end{array}$$

where *n* is the number of features. Additionally, we specified the value of the first quartile minus 1.5^*^interquartile range of the distribution as a threshold for low-coverage features. A low-coverage feature was defined as a feature with a CH coverage value that lies below the threshold. Such features were identified for each pair of datasets.

To eliminate the influence of noisy data on the CH analysis, a density-based data clustering algorithm DBSCAN^2^ was applied to the data. Before each run of the CH algorithm, outliers were removed using the DBSCAN method.

### Machine learning method for classification of a dataset, including an algorithm to derive important features to differentiate two datasets

The prepared dataset was split into the train (80%) and test (20%) sets. The classification task was to distinguish patients between two hospitals. Four classifiers, namely Logistic Regression (LR), Random Forest (RF), Support Vector Machine (SVM), and AdaBoost (ADA) were used. Since the target label (hospital source identifier) was imbalanced, the “class weight” hyperparameter for LR, RF, and SVM was set to the “balanced” option. An optimal set of model hyperparameters were found using grid search with stratified 5-fold cross-validation on the train set. A ROC AUC score was used to evaluate the performance of the chosen model. Predictions on the test set were evaluated with ROC AUC, precision, recall, and F1 score metrics.

ML methods were trained twice. First, all features were used to train ML models. Second, features with low CH coverage were omitted from the analysis and ML models were retrained. This allowed judging, whether the discriminating ability of ML models was predominantly caused by different CHs of underlying data or by differences in underlying data distributions of corresponding hospitals.

### Python 3 modules used in this study and system requirements

In this study, the SciPy Python 3 spatial library with the Quickhull algorithm and the Delaunay class (Virtanen et al., 2020) was used for CH analysis. And the Scikit-learn implementations of ML classification methods (Pedregosa et al., 2011) were svm.SVC, linear_model. LogisticRegression, ensemble.RandomForestClassifier and ensemble.AdaBoostClassifier. CH and ML analysis was performed on the computational cluster of the RWTH Aachen University using 1 node with 40 cores, 2.66 GHz, 4 GB RAM. The longest runtime for the CH analysis was 16 min. The runtime for the ML script comprised 24 h. Analysis was tested as well on the 2018 quadcore laptop i7-8565U CPU @ 1.80 GHz × 8. It could be run as it is on most modern CPUs with minimal RAM usage. No GPU is required.

CH and ML methods used in this study are available as a python package “chgen”. Example scripts on how to use this package are available in the repository: https://git.rwth-aachen.de/jrc-combine/chgen.

## Results

### Application of CH analysis to each pair of hospitals

**Figure 2:**
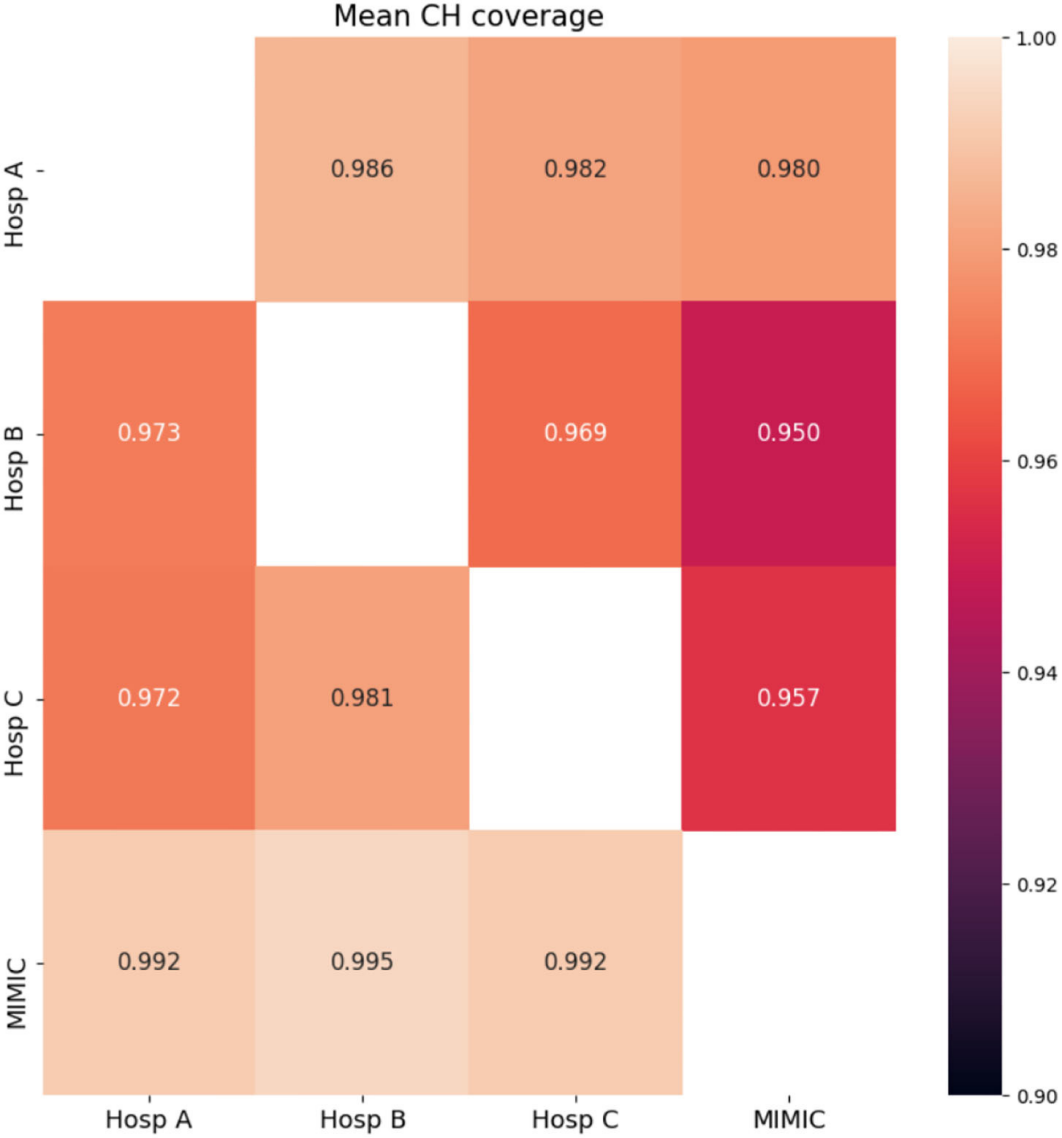
CH analysis results for data from four hospitals. Mean CH coverage over all features is shown. Rows-initial population, columns-population, whose CH is covered by the CH of the initial population.

Figure 2 shows the mean CH coverage for each pair of hospitals. For each German hospital, minimum coverage was found when data of the corresponding hospital were covering the MIMIC dataset (last column in Figure 2). However, that was not the case for the opposite situation. Maximum mean CH coverage was found for cases when MIMIC data covers data from German hospitals (last row in Figure 2).

Features with low CH coverage values were identified for each pair of hospitals. These features are shown in Table 3. The table is not symmetric since features with low coverage values when the first hospital's data cloud is covering the second one may be different from features in the opposite coverage situation. Results of the mean CH coverage are accompanied by the number of features with low CH coverage in each case of the datasets' comparison. For each German hospital, a maximum number of such features was found when data of German hospitals were covering the MIMIC dataset (3 or 2 features correspondingly, last column in Table 3). CH coverages for all features in the case of MIMIC coverage are given in Supplementary Table S1.

**Table 3:** Lists of parameters with low CH intersections for all pairs of hospitals.

	**Hosp A**	**Hosp B**	**Hosp C**	**MIMIC**
Hosp A	-	Tidal volume, PEEP	Tidal volume	PaO_2_, Tidal volume, PTT
Hosp B	PaO_2_, Respiratory rate	-	Bicarbonate arterial, Respiratory rate, PTT	PaO_2_, Bicarbonate arterial, PTT
Hosp C	FiO_2_, PEEP	-	-	PaO_2_, PEEP
MIMIC	FiO_2_, Lactate arterial	Lactate arterial	Lactate arterial	-

Rows-initial population, columns-population, CH of which is covered by the CH of the initial population.

### Application of ML routines for classification of the hospital

Results of the application of ML routines to classify the hospital for every pair of hospitals are shown in Supplementary Figure S1A. Results of the ADA method are shown, as it gained the highest performance in terms of ROC AUC in all cases. In each pair of hospitals, the hospital where the patient samples were derived from could be almost perfectly classified (ROC AUC ≥ 0.94). The best separation was obtained between the MIMIC cohort and German hospitals. German hospitals looked more alike to classifiers. The worst separation was observed between Hosp B and Hosp C.

After the exclusion of the features with low CH coverage values, and retraining with the best-performing ML classifiers, the largest ROC AUCs were still observed between the MIMIC cohort and German hospitals (see Supplementary Figure S1B).

### Use case example: Classification for ARDS on the first day of treatment in ICU

**Figure 3:**
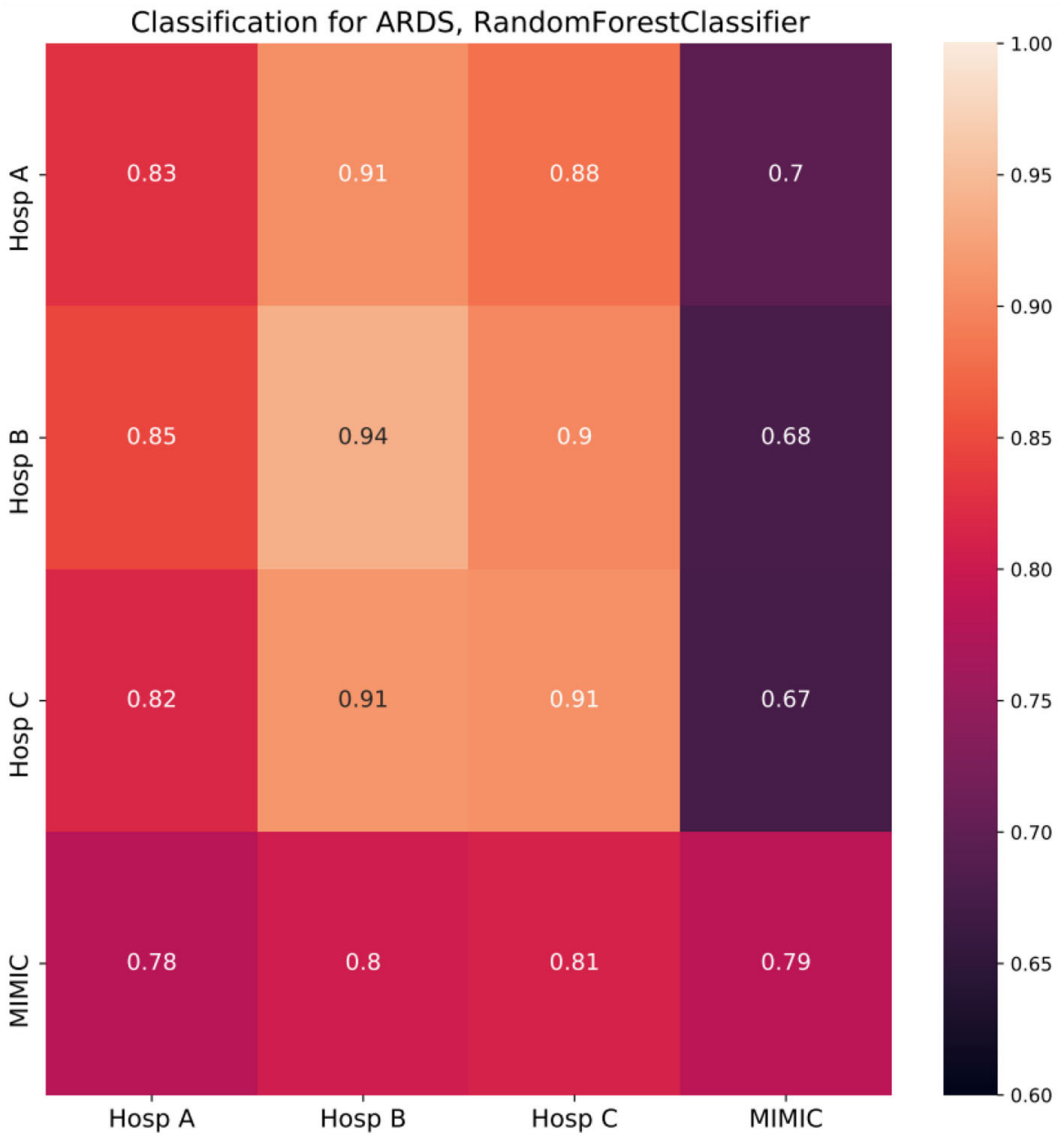
Random forest classifier classification results (cross-prediction matrix) for ARDS on the first day in ICU. RF trained in each of the four hospitals (row name) and applied in each of the four hospitals (column name). Diagonal cells represent the performance of specialized models which were trained and tested in the same hospital. Non-diagonal cells represent the performance of such models once they are applied in other hospitals and reflect ability of a model to generalize to the unseen population of another hospital. Twenty-one features common for all four hospitals were used to build corresponding RF models. Performance is depicted in terms of ROC AUC.

The results of the classification task are shown in Figure 3. Diagonal cells represent the performance of a specialized model which was trained and tested in the same hospital. The performance of specialized models strongly differed among hospitals under consideration, with the lowest ROC AUC of 0.79 in MIMIC and the highest of 0.94 in Hosp B. To test the generalization ability of developed models, they were tested on other unseen datasets, i.e., other hospitals (non-diagonal cells).

If the population of the new hospital is similar to or more homogeneous than the one of the original hospitals concerning the condition under consideration, the performance of the model will stay on a similar level or can be even higher than in the original hospital. However, if the population differs from the original one, performance will be impaired. For each specialized model trained in German hospitals the largest drop in performance was observed when the respective model was applied in the MIMIC dataset with the strongest drop of 0.26 for a model trained in Hosp B. Overall, models developed in Germany, showed impaired performance compared to the specialized MIMIC model. The opposite was not the case, as the MIMIC model showed similar performance in German hospitals to the performance in the original cohort.

## Discussion

“Internal” model performance on structurally similar, previously unseen data, gathered from the same source used for model training, can be contrasted with “external” model performance on new, previously unseen data from other sources. ML models perform worse in external cohorts due to several reasons such as different protocols, confounding variables, or heterogeneous populations (Cabitza et al., 2017; Zech et al., 2018; Martensson et al., 2020; Goncalves et al., 2021). Moreover, medical data can be biased by a variety of factors such as admission policies, hospital treatment protocols, country-specific guidelines, clinician discretion, healthcare economy, etc. Furthermore, labeling or coding criteria of a certain disease or syndrome and treatment guidelines evolve with time (Kunze et al., 2020). Since ML models for healthcare are predominantly developed on retrospective data, it remains unclear how the performance of such models is affected by the temporal separation of the target group even within one hospital.

Similarly, model reproducibility and model transportability have distinct objectives (Justice et al., 1999). While reproducibility focuses on the performance of the model in the same target population, transportability refers to performance in different but related source populations. Nevertheless, the closeness of this relationship between populations must be ascertained to achieve valid results of external validation. The performance will be poor in a sample that is too different from the data used for development. Conversely, a test sample that is too similar will overestimate the predictive performance showing reproducibility rather than transportability. To address these different aspects, an elaborate validation approach as described by Debray et al. seems necessary. They recommend the examination of the validation datasets in the first step to ensure adequate relatedness using a case-mix of a dataset and subsequent evaluation of the model with respect to the perceived relatedness (Debray et al., 2015).

In this study, we have introduced another method for population comparison and assessment of a model's generalizability. First, it estimates the similarity of the underlying populations in terms of mean CH coverage. Second, it estimates the differences in datasets in terms of underlying data distributions. These two tasks are accomplished by the application of 2 methods–first the CH analysis and followed by the ML classifiers.

During the application of the pipeline on the datasets obtained from 4 hospitals, we found that there were significant differences in CH coverage among pairs of hospitals. The lowest CH coverages for each of the German hospitals were observed when the MIMIC dataset was covered by data obtained from the corresponding hospital. However, in the opposite case i.e., Hosp A/Hosp B/Hosp C covered by MIMIC, the coverages were large. This shows that Hosp B/Hosp C and to a lesser extent Hosp A represented a part of the data space, spanned by data of MIMIC. In other words, data from German hospitals comprised, in greater or lesser proportions, parts of the MIMIC data cloud.

All four datasets exhibited differences in underlying data distributions. Once trained, ML classifiers were able to distinguish data coming from different sources with ROC AUC larger than 0.94, suggesting nearly perfect identification of the hospital from where the patient data originated from. After the omission of features with low CH coverages, the performance of retrained models dropped. However, the performance of models distinguishing MIMIC from German hospitals was still largely supporting the finding, that the MIMIC dataset significantly differed from German hospitals.

To demonstrate that our pipeline can be used to assess the generalization ability of ML models, we considered a use case of classification for the first day of ARDS data. A specialized model was trained for each of the four hospitals' data. Then it was applied to unseen hospital data and the performance of the model on the original data was compared to those of the new data. We observed 2 clusters of datasets, namely German hospitals and MIMIC. Models developed for German hospitals' data exhibited the largest drop in performance once applied to MIMIC. That was not the case in the opposite situation, i.e., application of the MIMIC model to German hospitals data, where almost no drops were observed. CH analysis fully supported these findings. First, for each of the German hospitals, the lowest CH coverages were observed when the MIMIC dataset was covered by data from corresponding hospitals suggesting the impaired performance of models developed in German hospitals and applied in MIMIC. Second, mean CH coverages of German datasets by MIMIC data were found close to 1, suggesting full CH coverage and thus, the absence of limitations for generalization.

Moreover, smaller drops in performance were observed when models developed on data from Hosp B or Hosp C were applied to data from Hosp A. This is in line with corresponding CH coverages (Hosp A by Hosp B/Hosp C), which are in the medium range. Interestingly, when models, developed in Hosp A or MIMIC were applied in Hosp B or Hosp C we did not observe any drop in performance, but even a slight increase. It could be the case if the population of the new hospital is similar to or more homogeneous than the one of the original hospitals concerning the condition under consideration. In our case, it would mean, that fewer non-ARDS patients with low Horowitz index are present in Hosp B/C compared to Hosp A/MIMIC. On the other hand, the necessary condition for the proper generalization, in this case, is satisfied by the fact, that CH coverages of Hosp B/C by Hosp A/MIMIC are among the largest in our study. Overall, the results of cross-prediction for ARDS were found to be in accordance with the results of the CH analysis of corresponding datasets.

Application of ML routines for classification for a hospital also supported the finding, which suggests that the MIMIC data significantly differed from German datasets, as the best separation with ROC AUCs > 0.99 was obtained between the MIMIC cohort and German hospitals. Nearly perfect separation was still possible after the exclusion of features with low CH coverage. This result indicated that the MIMIC cohort is not only less covered by German data, but exhibits diverging underlying data distributions once compared to German hospitals. However, while ML methods indicated, that there were significant differences in underlying distributions and performance of a model could be impaired, they did not point in the direction of proper or poor generalization, i.e., models trained in dataset A and applied in dataset B and vice versa. This constitutes an advantage of the CH method, as it is originally asymmetric and allows to assessment direction of impaired generalization. Moreover, the CH assessment is universal and does not depend on the particular ML classification method.

However, there could be multiple other reasons for such strong discrepancies in models' performance. First, some of the features with low CH coverage (PEEP, FiO_2_) belong to parameters, which are set by physicians in the ICU, thus suggesting different treatment strategies in underlying hospitals. Second, diverging ARDS labeling criteria (ICD-10 in Germany vs. ICD-9 in MIMIC) might contribute to label uncertainty in ARDS classification. Finally, the timespans of data collection overlap only partially. MIMIC data were collected between 2001 and 2012, Hosp A data between 2009 and 2019. Data from Hosp B and Hosp C were collected after 2012. This is relevant since in 2012 the American European Consensus Conference (AECC) definition of ARDS changed to the currently accepted Berlin definition (Kunze et al., 2020).

Nevertheless, the main observation is valid regardless of particular ARDS labeling: MIMIC data do significantly differ from all three other hospitals in this study. Given that this database is considered nearly a gold standard of open ICU databases, an external validation for models developed on this database is absolutely necessary. In the best case, a special pipeline for the assessment of the transferability of trained models should be included in the data preparation step before a model development, so that generated models might exhibit significantly better performance.

Our study has other limitations that have to be considered. It is known that CH analysis is very sensitive to noise in the data (Worton, 1995). To eliminate the influence of noisy data on the convex hull analysis, a density-based data clustering algorithm DBSCAN (Schubert et al., 2017) was applied to the data so that during each run of the CH algorithm, outliers were removed using the DBSCAN method. Additionally, to increase the robustness of the CH analysis results, each CH analysis execution was averaged over 100 runs with bootstrapped data. Another potential weakness of our study design is that imputation was done without taking into account multidimensional parameter distribution. However, this could not significantly influence the main conclusions on differentiating parameters in this study, as we specifically have chosen patients with charted data of the main parameters important in the ARDS state: PaO_2_, FiO_2_, and PEEP. Another important question is how to define cutoff values between good and bad performance for both CH and ML analysis. We estimated CH coverages between train and test sets for the same hospital (see Supplementary Table S2). These can be used as benchmarks for CH intersections for reasonable generalization. However, these also differed among hospitals, but here a clear correlation with the sample size of the cohort was observed. For instance, in Hosp C, a test set of 202 patients was covered by a train set of 810 patients. Therefore, the estimates for proper CH coverage should also depend on the sample size under consideration. For large datasets (Hosp A/MIMIC), where test set sizes were comparable to the sizes of smaller datasets in the study (Hosp C) they comprise 0.987/0.972. For ML routines, there is no rule of thumb to define minimum ROC AUC to judge whether hospitals cannot be distinguished. Usually, values of ROC AUC < 0.7 are considered poor discrimination performance.

Additionally, sample size can potentially be a factor, while considering convex hull intersections and machine learning results. However, there are some pieces of evidence, that this is at least not a dominant factor for generalization differences. First, models developed in small cohorts of Hosp B/Hosp C for ARDS classification deliver similar performance in Hosp A, as a specialized model of that hospital. Second, the model developed in Hosp A has a high generalization error in MIMIC (0.13), but a model developed in MIMIC shows the opposite behavior in Hosp A having a small generalization error (0.01). Third, a model developed in the smallest Hosp C does not exhibit any generalization error in a dataset of completely different Hosp B. Therefore, we are of the strong opinion, that different sample sizes in underlying hospitals cannot explain such strong discrepancies in models' performance in different hospitals.

Another important remark is that as the dimension of a dataset grows, then a trained ML model will almost always lay in the extrapolation range once applied to unseen data (Balestriero et al., 2021). This is a consequence of the curse of dimensionality and has to be considered in all ML applications and especially in deep learning where models are dealing with hundreds or thousands of features. However, ML models that utilize continuous time series data and are applied in real healthcare settings usually require a degree of interpretability and therefore contain a limited number of features (Chen et al., 2019). This was also the case in our study, where the number of features did not exceed 30.

## Conclusions

Currently, with the ever-growing number of AI and ML models in healthcare, there is a huge challenge in the translation of such models into clinical practice. In healthcare, new data are often different from those used in the training of the model. To achieve a clinical implementation, a model must be able to perform with sufficient accuracy on previously unseen data. Hospitals may have different policies, guidelines, or protocols, but even within one hospital, guidelines could change over time causing altering patients' responses.

Therefore, the validation of developed models before a potential application at the bedside plays a key role in translation research. With the pipeline introduced in this study, we contribute to the solution of this issue. Given the training data and a retrospective dataset from a hospital, where the model is intended to be used, we can judge the generalization ability in another hospital. On the use case of classification for the first day ARDS, we showed that the strongest drop in performance is associated with the poor intersection of convex hulls of corresponding hospitals and with differences in underlying data distributions. Therefore, we suggest the application of our pipeline as a first tool to assess the transferability of trained models.

Based on our analysis of four different hospital datasets, we conclude that datasets from different hospitals represent heterogeneous data sources and the transfer from one database to another should be performed with care to avoid implications during real-world applications of the developed models. Further research is needed to develop methods for the adaptation of ML models to new hospitals. In addition, more work should be aimed at the creation of gold-standard datasets that are large and diverse with data from varied application sites.

## Data availability statement

The original contributions presented in the study are included in the article/Supplementary material, further inquiries can be directed to the corresponding author.

## Author contributions

HM, SF, KS, RP, and KN worked on data acquisition and harmonization. KS, MS, and KN developed and implemented CH analysis scripts. KS, JSB, and KN developed and implemented ML routines. KS, JSB, and AS designed the research, performed analysis, analyzed the patient data, and developed the ARDS prediction model. SF gave medical advice during the development of the pipeline. SF, GM, and JB interpreted the results from a medical perspective. KS, JSB, SF, and AS wrote the manuscript. All authors read and approved the final manuscript.

## Funding

This publication of the SMITH consortium was supported by the German Federal Ministry of Education and Research, Grant Nos. 01ZZ1803B, 01ZZ1803I, and 01ZZ1803M.

## Conflict of interest

HM is an employee of Bayer AG, Germany. HM has stock ownership with Bayer AG, Germany. The remaining authors declare that the research was conducted in the absence of any commercial or financial relationships that could be construed as a potential conflict of interest.

## Publisher's note

All claims expressed in this article are solely those of the authors and do not necessarily represent those of their affiliated organizations, or those of the publisher, the editors and the reviewers. Any product that may be evaluated in this article, or claim that may be made by its manufacturer, is not guaranteed or endorsed by the publisher.

## Footnotes

1 https://github.com/MIT-LCP/mimic-code/blob/62102b08040ac5db96af7922db8d7832ce30a813/etc/ventilation-durations.sql

2 https://scikit-learn.org/stable/modules/generated/sklearn.cluster.DBSCAN.html

## Supplementary material

Supplementary Figure S1 ROC AUC for classification for a hospital. **(A)**: Performance of an ML learning algorithm for classification for a hospital. **(B)**: Performance of an ML learning algorithm for classification for a hospital after removal of features with low CH coverage values. Numbers in cells reflect the ROC AUC of the classifier trained to separate between hospital 1 (row name) and hospital 2 (column name).

Supplementary List S1 Diagnostic parameters used in this study. Overall, 54 diagnostic parameters routinely assessed in the ICU were used in this study. Additionally, 6 biometric parameters were used.

Supplementary List S2 List of parameters used for classification ARDS on the first day in ICU.

Supplementary Table S1 CH coverages for all features. MIMIC data covered by other hospitals.

Supplementary Table S2 CH coverage of the test set by the train set in the same hospital, where ML models were developed.

## Abbreviations

ARDS: Acute respiratory distress syndrome
CH: Convex hull
ICU: Intensive care unit
FiO_2_: Fraction of inspired oxygen
MV: Mechanical ventilation
PaO_2_: Arterial partial pressure of oxygen
PEEP: Positive end-expiratory pressure
ROC AUC: Area under receiver operating characteristic curve.
