# Supplementary figures and images for: Application of convex hull analysis for the evaluation of data heterogeneity between patient populations of different origin and implications of hospital bias in downstream machine-learning-based data processing: A comparison of 4 critical-care patient datasets
Source: Front Big Data. 2022 Oct 31;5:603429. doi: 10.3389/fdata.2022.603429 (PMC9659720; doi:10.3389/fdata.2022.603429)

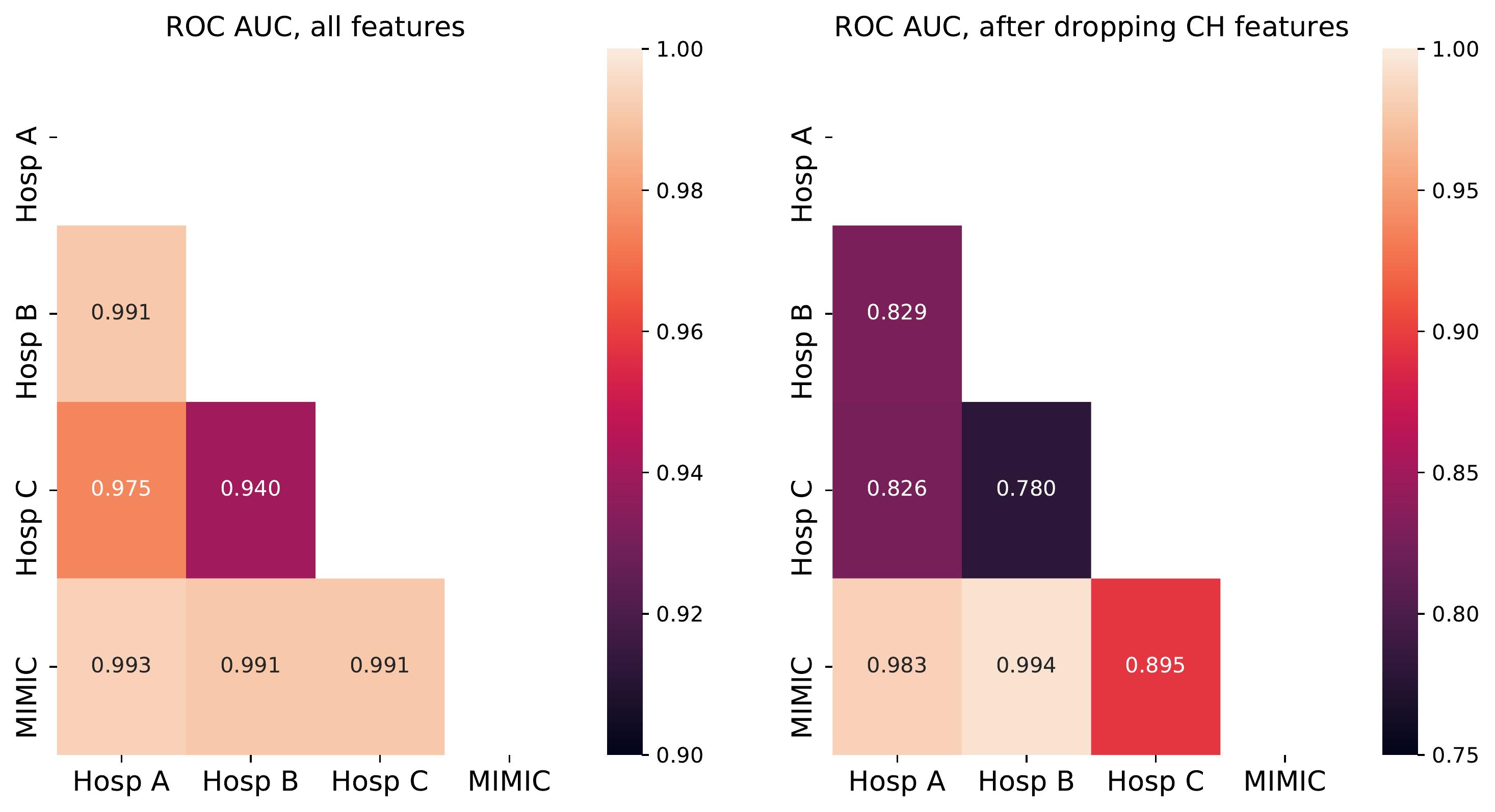

Supplement: Supplementary Figure S1 — ROC AUC for classification for a hospital. (A): Performance of an ML learning algorithm for classification for a hospital. (B): Performance of an ML learning algorithm for classification for a hospital after removal of features with low CH coverage values. Numbers in cells reflect the ROC AUC of the classifier trained to separate between hospital 1 (row name) and hospital 2 (column name). [file Image_1.JPEG]
