# Supplementary material for: Application of convex hull analysis for the evaluation of data heterogeneity between patient populations of different origin and implications of hospital bias in downstream machine-learning-based data processing: A comparison of 4 critical-care patient datasets
Source: Front Big Data. 2022 Oct 31;5:603429. doi: 10.3389/fdata.2022.603429 (PMC9659720; doi:10.3389/fdata.2022.603429)
Supplement: Supplementary List S1 — Diagnostic parameters used in this study. Overall, 54 diagnostic parameters routinely assessed in the ICU were used in this study. Additionally, 6 biometric parameters were used. [file Data_Sheet_1.docx]

**Supplementary List 1.** Diagnostic parameters used in this study. Overall, 54 diagnostic parameters routinely assessed in the ICU were used in this study. Additionally, 6 biometric parameters were used.

Vital signs:

- Heart rate

- Peripheral oxygen saturation (SpO2)

- Systolic arterial pressure (SAP)

- Mean arterial pressure (MAP)

- Diastolic arterial pressure (DAP)

- Central venous pressure

- Systolic pulmonary arterial pressure

- Mean pulmonary arterial pressure

- Diastolic pulmonary arterial pressure

- Body temperature

- 24h urine output

- 24h fluid balance

Ventilatory settings:

- Respiratory rate

- Respiratory rate (spontaneous)

- Tidal volume

- Tidal volume per ideal body weight

- Tidal volume (spontaneous)

- End-inspiratory pressure (P_EI_)

- Positive end-expiratory pressure (PEEP)

- Fraction of inspired oxygen (FiO_2_)

- Inspiration : Expiration ratio (I:E)

- Pulmonary compliance

- Inhaled nitric oxide

Blood gas analysis parameters:

- pH arterial

- PaCO_2_

- PaO_2_

- SaO_2_

- PaO_2_/FiO_2_ ratio (P/F ratio; Horovitz index)

- Base excess (arterial)

- Bicarbonate (arterial)

- Lactate (arterial)

- ScvO_2_

Laboratory parameters:

- Albumin

- Alanine transaminase (ALT)

- Amylase

- Aspartate transaminase (AST)

- Bilirubin

- Brain natriuretic peptide

- Creatine kinase

- Creatine kinase-MB

- Creatinine

- D-dimers

- Haematocrit

- Haemoglobin

- International normalized ratio (INR)

- Interleukin-6

- Lactate dehydrogenase

- Leukocytes

- Lipase

- Procalcitonin

- Platelets

- Partial thromboplastin time (PTT)

- Troponin

- Urea

Biometrics:

- Height

- Weight

- Age

- Gender

- ARDSnet ideal body weight (StdWeightARDS)

StdWeightARDS (Female) = 45.5 + 0.91 (Height [cm] - 152.4)

StdWeightARDS (Male) = 50.0 + 0.91 (Height [cm] - 152.4)
