# Supplementary material for: Application of convex hull analysis for the evaluation of data heterogeneity between patient populations of different origin and implications of hospital bias in downstream machine-learning-based data processing: A comparison of 4 critical-care patient datasets
Source: Front Big Data. 2022 Oct 31;5:603429. doi: 10.3389/fdata.2022.603429 (PMC9659720; doi:10.3389/fdata.2022.603429)
Supplement: Supplementary List S2 — List of parameters used for classification ARDS on the first day in ICU. [file Data_Sheet_2.docx]

**Supplementary List 2.** List of parameters used for classification ARDS on the first day in ICU.

Horowitz index, Respiratory rate, Tidal volume, FiO2, PEEP, PaO2, Lactate arterial, Bicarbonate arterial, SpO2, Heart rate, Leukocytes, Platelets, Urea, Creatinine, Haemoglobin, PTT, Height, Weight, Age, StdWeightARDS, Gender
